# Hope Through Tales: Through the Eyes of a Child of a Parent With Bipolar Disorder

**DOI:** 10.1111/jpm.70099

**Published:** 2026-01-23

**Authors:** Rachel Mariam Johnson

**Affiliations:** ^1^ Department of Speech & Hearing, Manipal College of Health Professions Manipal Academy of Higher Education Manipal Karnataka India

## Abstract

**Aim:**

To address the limited understanding of everyday family experiences of individuals diagnosed with bipolar disorder and to explore the effects of parental mood instability on young children and the possibility of early interventions to promote parent–child interaction and bonding.

**Background:**

One of the most challenging mental health disorders is widely acknowledged to be bipolar disorder. The everyday family experiences of individuals with this diagnosis, as well as their personal experiences, are far less well documented than the clinical and medical aspects. The effects of parental mood instability on their young children and the possibility of early interventions to promote parent–child interaction and bonding are poorly studied.

**Contribution to Existing Knowledge:**

This paper adds to the existing knowledge that a lot of familial instability and parenting issues arise with the diagnosis of bipolar disorder, which can cause a myriad of issues for the developing child, even at a young age. This paper also describes the use of shared book reading as a medium for a parent with bipolar disorder to spend time and bond with their child, even during their alternating mood states.

**Implications for Mental Health Nursing:**

Mental health nursing and other rehabilitation professionals could utilise these simple tasks to encourage the use of this method, thereby promoting parent–child bonding and interaction.

## Key Points/Highlights

- Bipolar disorder has a profound impact on everyday family life and parent–child relationships.
- Children's lived experiences of growing up with a parent with bipolar disorder remain under‐researched.
- Shared book reading may offer a simple and accessible way to promote parent–child bonding.
- Mental health nurses can support low‐intensity, relationship‐focused interventions within families affected by bipolar disorder.

## 1 Introduction

Bipolar disorder (BD) is a mental health disorder which manifests through cycles of depression and mania, one after the other, with brief periods of remission (American Psychiatric Association 2013). Since most of the symptoms of BD emerge only in late adolescence or adulthood, this causes significant interference in family life and stability (Bolton et al. 2021; Grande et al. 2016). Children of a parent with BD are the most affected since they are exposed to multiple factors like neglect, familial conflict, emotional instability and negative parenting (Koenders et al. 2020; Shalev et al. 2019; Stapp et al. 2020). As a result, these children often lose the bond with their parents and grow up in a dysfunctional home.

Shared book reading or simply reading together could pave a path for both child and parent to bond, even in such difficult circumstances where the parent has a mental illness. Encouraging and creating awareness of shared book reading as a simple means to engage with your child can help pave the way for stronger bonds between parent and child. Shared book reading has long been established as a way to foster bonding and interaction with children. It helps them build a strong attachment as well as provides them with enhanced academic success and literacy development (Clemens and Kegel 2020; Walker 2013). Literature on using shared book reading in mothers with mental health disorders like depression has proved that it also has a positive impact on their depression severity and, in many cases, has lowered scores (Kumar et al. 2016; Weisleder et al. 2019).

Though available literature is sparse, patterns and factors that affect families with BD have been identified (Anke et al. 2019). These studies report on the commonly seen challenges, but the real challenges and those that don't seem significant can only be reported by those who are experiencing them. Lived experiences provide insight from a different viewpoint, one that cannot be thoroughly tapped by any researcher who has not stood in the same shoes. Being a researcher and having the experience opens doors that can help families suffering from the same illness through feasible methods. As a researcher, I applied the most useful practice I found during my difficult childhood to help my interaction with my father, who was diagnosed with BD at the age of 21 years.

## 2 Experience

This is a recollection of my childhood as someone who was raised by a parent with bipolar disorder. I now reflect on these childhood memories through the eyes of an adult and would like to share my insight on what helped my relationship with my father and how the same could be applied to other families as well.

‘I think I have two dads. I could sense which one was there just by hearing one word from him. One dad is a smart, charming, happy‐go‐lucky, jolly fellow, full of wit and humour, ready to conquer the world. His eyes are bright, and he talks nineteen to a dozen. But I came to dread this. In two or three days, the same person may change into a rampant, raging individual who has to be rushed to the hospital. My other dad was the opposite. His face is dull and his eyes groggy, his body burdened with a load too heavy to bear. His twinkling laughter, excited talk, and humour fail to leave his body. He lies bundled up on the bed, not wanting to talk or listen to anybody. He faintly whispers, “I'm feeling low,”’ and goes back to his dark, gloomy world. My heart breaks for me. I'm too young to understand what my dad must be going through. All I know is that my dad was letting me down, did not want to play with me or did not want to do the things he promised me.

I ran to my father with my little doll. ‘Get up, get up… Papa!!!! See, dolly is waiting to play’. Papa grunted and rolled back to sleep, pulling the sheets tighter over his head. I shook him a couple of times. ‘Please, Papa… I'm bored. Please get up’, I cried, little tears forming in my eyes. He grunted out, ‘Later’, and fell asleep. I took my doll and went away to play alone, saddened that my Papa didn't want to play with me. I was used to this. It was always the two extremes. Either Papa would not get up from bed, or he would be flitting here and there excitedly. I was terrified of the other extreme. I would lock myself in my bedroom, away from all the fights and shouts. I used to hear him proclaim that he was God, throw things around and shout, and had to be cornered by security personnel, put into a barred ward, and admitted. Periods of time used to go with hospitalisations and heavy medications. These were the times when I kept my distance. I used to observe from afar and visit my drowsy father while in the hospital. I still loved him to bits. Just when I thought he was back to himself, he went into the other extreme of depression. I remembered my father locking himself up and my mother banging on the door, pleading to open it and not do anything bad. I remembered my father talking about dying and death, and remembered how scared I was thinking of losing my father. Then came the rare weeks when everything would be normal. I loved those rare instances. We were the happiest family in the world. We used to play, go for outings and talk to the dozen until the cycle repeated. As I grew up, I found fewer and fewer of these rare weeks, as my father's illness, too, grew increasingly severe. Our bond had decreased, and I found it so hard to bond with my father.

Mom told me that it's an illness and not his fault. I was not entirely sure what she meant when she told me it was called bipolar disorder. I knew that it made my Papa change and that it felt like a huge storm enveloped our little home. I didn't know what to expect every morning. I was afraid I would trigger the storm somehow. I tried to be extra good because I didn't want him to yell, but sometimes, when he's in one of his sad moods, he doesn't even notice I'm there. When he's happy, we have the best time ever; we go on walks, play Monopoly and Scrabble, and joke and laugh together, but at night, I still didn't know which dad I would wake up to. I have two worlds within my home. One world was full of noise and laughter, and the other world was quiet, dark, and still.

One fine day, I found an outlet. My father and I loved reading. I made a routine. Irrespective of Papa's mood, I would sit with my father after our dinner and make him read a funny excerpt from the paper or a storybook. At first, especially when he was depressed, he would make all sorts of excuses to go to sleep, but when he saw I was insistent, he began making it a routine. I curled up next to him and listened to him read. Slowly, his voice changed; he became more invested in what he was reading, and he smiled at the funny jokes within. I cracked some of my own, too. Except when he was in a manic state, we continued this routine for a long time. I learned that even when my dad couldn't be the happy dad I always remembered, we could still find ways to be together. The stories we shared made us feel like we were on the same team, no matter how big or small the storm in his heart was. In those quiet moments, surrounded by pages and pictures, I realised that sometimes, the best way to connect with someone is not by asking them to be something they're not but by simply sharing something that brings peace. And for us, those stories became the bridge between the two dads—the one full of light and the one who needed time to heal. I feel this was one of the factors that led to my strong, unbreakable bond with my father. One that I cherish still, long after he is gone.

How can we bring about a sense of normalcy within this world? It has been studied that maximising the use of remission or stable states between mood swings could benefit both the patient and their caretaker or family. It never occurs to us that a small child might be the one most affected. What if I told you that even something as simple as sitting and reading together could make a change for the child and the affected parent? World Bipolar Day is celebrated on March 30th every year. Every year, I see people post awareness videos and write‐ups, but are you aware of what happens within? Can you ever imagine yourself in the shoes of someone living with a severe mental health disorder? The highs and lows they go through? Now imagine yourself as someone beside that person for the rest of your life. Difficult, isn't it?

## 3 Future Research and Implications

I call for further research that addresses basic issues like this. It may not be something significant, but it can have a profound impact on the life of the affected parent and child. Interventions which promote interaction and bonding using simple methods could be developed.

Mental health nurses, being a point of constant contact for the patients, could be the ones to refer patients they feel could benefit from such interventions. They could help pave the way for referrals, as well as provide constant encouragement for the patient and child. They could even take on the roles for initial assessments of interaction and bonding, and report their observations about the parent and child, which could help tailor the interventions according to their needs.

Rehabilitation professionals, such as speech‐language pathologists, could take on these responsibilities, including providing and designing interventions tailored specifically to the needs of the parent and child.

## 4 Conclusion

This is an instance where a child was exposed to her father's bipolar disorder. For a small child, its parents are the world, and to have its world turned upside down due to this illness is something we all need to think about. Shared book reading (SBR) is a simple, direct task that can be utilised to promote family time, reading, bonding and interaction within families or between parents and children. This will give the dyad an outlet to bond and further enhance their attachment.

## Funding

The author has nothing to report.

## Conflicts of Interest

The author declares no conflicts of interest.

## Data Availability

Data sharing not applicable to this article as no datasets were generated or analysed during the current study.
